# Transactions of the Medical Association of Southern Central New York

**Published:** 1854-11

**Authors:** 


Art. IV.—*Transactions of the Medical Association of Southern Central New York*, at the eighth annual meeting, held at Homer, June, 1854.

The Transactions of the Medical Associations of our country possess much interest, not only for the valuable information which they bring forth, but as marking the activity of medical men in reference to the profession. They afford evidence of life. They denote progress, and in this point of view are highly important. We propose making extracts from the volume of Transactions before us, which contains an unusual variety of matter. We pass over the President's Address, and the Essay on Medical Education, to notice papers of a more practical character. One of these is an essay on Opium and the sulphate of Quinine as remedial agents. The author, Dr. Nivison, presents the following views in reference to their use in fever:

"We shall usually find, when called upon to treat a case of Idiopathic Fever, without much reference to its type, or indeed the stage of its progress, very many of those symptoms which indicate a discordant state of the nervous system. There is either prostration, or pain, or delirium, or what is more disagreeable than either, and which I know not that any language can describe; it is neither restlessness, nor suffocation, nor jactitation, nor anything else in particular; but rather a componnd of all manner of disagreeable sensations. Either some one or more of these conditions, (unless, indeed, the patient should chance to be in the comatose stage of a congestion,) will probably be present.

Our first object in the treatment will therefore be, to restore the tone and regulate the action of the nervous system. From two to four grains of Quinine, with a grain or two of Opium, repeated once in from four to six hours, will usually soon accomplish this. The patient soon feels better, the countenance becomes more animated, the pulse approaches the healthy standard, the circulation is comparatively equalized, and the skin usually moistened. If we can secure and maintain this state of things, which is usually not difficult, we have the case under control. The next important step, is to look to any complications that may exist. And if we have carefully noted the symptoms before the Opium and Quinine took effect, and compare them with those that still remain, we shall probably find that very many of those that indicated local congestion or inflammation have disappeared. We have already put the general system in the best possible condition for the *vis medicatrix naturæ* to exert her latent powers. But obstacles may exist, to the removal of which her utmost efforts are inadequate. If such we find to be the case, there is an urgent demand for interference on our part, and we shall perhaps be surprised to find the greater facility with which we can now accomplish our purpose, than before the effect of the Opium and Quinine had been obtained.

"Now, that we have enlisted Nature's forces in our service, we will find a mustard plaster more potent in removing some local congestion than the entire armament of cups, leeches, blisters, &c., would have been before. Two or three blue pills, followed with a sedlitz powder, or a dose of castor oil, will more effectually arouse the liver, and evacuate the bowels, than half a drachm of calomel would previously have done; and this, too, without violence to the mucous coat of the stomach and bowels. Indeed, we think if this system of treatment were more generally adopted, we should have very much less to charge to the account of gastro-cuteritis as the cause of fevers.

"To secure and maintain the tone of the nervous system, and to treat any local complications as they arise, we think comprises nearly our whole duty in the treatment of fevers; all else we may safely leave to the recuperative powers of nature. If the treatment be commenced early, we shall usually cure the disease before any considerable local disturbance arises. If not till a later period though we may not be able to arrest the disease at once, we may still shorten its duration. We think the ancient dogma, that 'we can guide a fever, but cannot cure it,' to be substantially incorrect. It is but just, however, to remark that a limited number of cases have fallen under our observation, in which the function of innervation partially refused to yield to the influence of Opium, Quinine or anything else, and the fever continued for a number of weeks. But these cases are so rare as to form the exceptions and not the rule, and even in the cases referred to, it is possible some concealed enemy was lurking beneath the surface, and possibly if the Quinine had been used in larger doses the results would have been more gratifying. With the heroic doses of modern times we confess we have had no experience, though we sometimes give 20 or 30 grains during the twenty-four hours.

"In regard to Opium, we consider it almost invaluable in the treatment of fevers; yet it is obvious that so potent a remedy must be used with extreme care. Though we may remark that in combination with Quinine, the system will tolerate it infinitely better than when given alone. Many patients that, from constitutional idiosyncrasy cannot use it at all when given alone, in combination with Quinine will take it without inconvenience. In general, in giving Opium in fevers, we may content ourselves with the exhilarating effect; but if local inflammation exist, they will modify the febrile action, and we may then approach its sedative effects. An occasional inconvenience from its use is to produce copious perspirations. We must here diminish the dose, or if need be, for the time being, suspend it altogether. In the purely intermittent forms of fever, we consider it less important than in the continued. The Quinine we think equally useful in all."

The following essay will be read with interest:

"*An Essay on the Depraved General Health sometimes occurring before Parturition, and its consequences.* By. R. O. Crandall, M. D., Waverly, New York.—The object of this paper is to present a few thoughts, suggested by a limited experience, upon one of the morbid conditions occasionally connected with, and modifying the puerperal state; to wit: a depraved general health, before parturition, and its consequences.

"We may premise here, that it will not be difficult to distinguish this mixed disease from the class of puerperal diseases since we are familiar with *their* more prominent symptoms, acquired from the text-books, if not from personal experience. On the other hand, the disease in question has been considered of so little importance as hardly to merit a passing notice.

"From my own experience, however, with general principles as the only guide to treatment, it appears to demand attentive consideration. The derangement of the general health as applicable to this subject, the following train of symptoms will sufficiently illustrate:

"The patient is a female, aged 36 years, the mother of three children, residing upon a farm on the bank of the Chemung river, and in a neighborhood where bilious remittent fever prevails. She complains of having been unwell for nearly a year past; did not think herself in need of medical treatment, but thought it would wear off. She had not given up to it—in fact, she had performed her ordinary household duties. Latterly it required an extra effort and the lightest labor induces fatigue.

"The first tangible sign of illness she can recollect, is a feeling of lassitude; then loss of, or depraved appetite, and a desire to avoid physical and mental exertion. Next notices a dull pain in the side, which, after a few days' continuance, extends along the back to the shoulder blade, and had been persistent. Pain also in the head in the after part of the day, and constipation of the bowels, alternating with diarrhœa. There are pains in the back and hips, frequent palpitation of the heart, and acidity of the stomach. The face becomes pale, or assumes a yellowish hue. The body is flaccid, and likewise pale, the feet are affected with edematous swellings. The patient's sleep is disturbed, and she is irritable and desponding. The general system confesses to an earnest sympathy for its component parts.

"Thus much in explanation of the condition referred to. To proceed, as near as may be, into the order of events: Sometime within the year, in which the foregoing train of symptoms were developed, or perhaps within the last six months, the patient became pregnant. She has not deemed it necessary as yet to consult her physician.

"Gestation now furnishes to her mind a satisfactory explanation of the cause of her ill health. She anticipates ill health for months to come, and flatters herself that her confinement will completely restore her. Thus the case is neglected and permitted to progress uninterruptedly, for several months, because the consequences of neglect under these peculiar circumstances are not duly appreciated.

"The legitimate consequence, is an additional degree of derangement of the local organs, and of the general system. Since, the same causes remain, continue in operation, and are not materially modified by utero gestation.

"In the very grave cases in addition to the symptoms already described, there is serous effusion into one or more of the cavities of the body. The effusion usually commences with edematous swellings of the lower extremities succeeded by ascites and hydrothorax. The constitutional symptoms are loss or want of appetite, great debility, with the quick, weak fluttering and irregular pulse of palpitation, indicating a state of high nervous irritability.

"In these cases, it becomes a matter of serious doubt whether the patient will survive to experience the pangs of labor, nor do they always. The great majority of cases, however, pass through parturition with little or no apprehension of danger, either on the part of the patient or physician, unless, happily, the wits of the latter have been sharpened by personal experience in similar cases.

"The parturient state is modified in a greater or less degree, varying with the previous constitutional dyscrasy. Serous effusion to any considerable extent, adds greatly to the distress of the patient. There is a relaxed state of the muscular system, and a consequent diminution of resistance. There is also a feeble contractile or expulsive power; and the capacity for endurance is proportionable small, but delivery is accomplished quite as speedily, and with no more suffering than in ordinary cases.

"The patient has now entered upon the puerperal state, very many sources of danger have been removed, but the effects of perhaps protracted suffering, of violent pain, of mental alarm, and of 'what may be termed the shock of parturition,' have an important bearing upon the case, and are to enter into its consideration.

"The patient finds herself very much relieved; the mental and physical anguish is past, and she is encouraged with the hope of 'a good getting up.' Ordinarily, the appearances are improved for a longer or shorter period; and the physician too, unless extremely cautious, indulges hopes which the convictions of his better judgment will not sustain.

"But if it is one of the cases in which effusion has taken place, other than mere œdema, there is increased prostration of strength, and you have, within forty-eight hours, a decided aggravation of all the symptoms. There is febrile action, indicated by heat of skin, and thirst; but the external surface remains pale or sallow; the mucous membrane of the mouth and tongue is likewise pale; there appears to be an insufficient proportion of coloring matter in the blood to produce even a decided flush of redness. All the cavities of the body are rapidly filling with serum; the expression of the countenance is not indicative of pain, but of great anxiety in consequence of the constant and distressing palpitation, and a sensation of impending suffocation. The bowels are confined, the urine scanty and highly colored. The patient cannot assume the horizontal position, but reclines with the head and shoulders elevated. The appearances all point towards a fatal termination. The termination may be either speedily fatal, protracted to another pregnancy, or postponed indefinitely, depending very much upon the precise course of treatment adopted and carried out, though the great majority, I believe have a speedy termination.

"In the cases unattended with dropsical effusion, the constitutional disturbance is frequently comparatively slight; the same peculiar appearance of the mucous membrane, and of the skin, confined bowels, and occasional palpitations. Taken as a whole, in connexion with the puerperal state, the symptoms do not appear to indicate any cause for serious apprehension, with the exception perhaps, of the general anæmia, as denoted by the paleness of the general surface, and more especially of the prolabia, the internal surface of the cheeks, the gums and the tongue.

"Patients under these circumstances are occasionally discharged within a week after confinement, with the impression that they will soon get up, and do not require farther treatment. The patient, however, continues, although imperceptibly, to fail in strength, the characteristic paleness of the mucous membrane becomes more striking, the desire for food is wanting, or failing, but there is no complaint of pain. The general expression does not indicate physical suffering; there is no febrile excitement. There is almost an entire absence of *positive* symptoms of disease, but the patient sinks about the third week after confinement.

"With the pathological appearances I am not familiar. I believe them to be very similar to those of anæmia. In some of the cases, organic disease of the stomach, liver and spleen, in others no appreciable appearances to account for the result—merely a decided general paleness of the tissues. The course of treatment apparently indicated, and that generally practised, so far as my observation has extended in this direction, is mild alteratives and tonics, such as mercury and iron, strict attention to the state of the bowels, to diet, air, &c. Opiates are frequently indispensable, to allay nervous excitability. My professional neighbors believe these cases almost uniformly fatal. Dr. Hoyt of Athens, Pa., gave me some account of seven cases, all of which were fatal.

"In my own practice I have varied this course of treatment by administering daily, or every other day, according to circumstances, a full cathartic dose of calomel, notwithstanding the extreme debility, until three or four doses have been taken, or until a change can be detected in the appearance of the mucous membrane. A thorough action of this agent, instead of increasing the exhaustion, has the effect of a powerful tonic.

"The patient expresses her surprise, not only that she lived through the operation, but that she really feels stronger; and such is the case. After the morbid actions are once interrupted, or rather changed, the mild tonic and alterative course is the appropriate one. I have very much encouragement to persist in this modified treatment."

The subjoined case is instructive:

"*Hypochondriasis resulting in death.* Reported by Dr. T. H. Squire.—Henry Potter, Esq., was a man fifty years of age, spare in person, and of melancholy temperament. Of his early history and health I have no definite knowledge. For the last few years, his habits have been sedentary. Acting as Justice of the Peace, and County Clerk, his mind has been taxed by monotonous cases, whilst his physical frame has been comparatively at rest. To the fatiguing duties of his office were added the cares of a large family, dependent mainly upon him for their support. This state of things made no sensible impression on his health, however, so long as the immediate future presented no worse prospects; but when the expiration of his term of office drew near, apprehensions of coming adversity took possession of his thoughts, and he grew more melancholy and desponding. His office finally expired. His features grew long, his countenance assumed a darker shade, and he was well nigh sick a-bed. Just then he obtained a clerkship in the Post-office, where his labors occupied the whole day, and often trespassed much upon the night. Soon this situation was taken from him, and then, being unemployed, he sat down in the midst of his family, and gave way to the most gloomy forebodings. His appetite left him. For several days he paced his floor in moody silence. Evidence of mental delusion then came on. He fancied himself not only poor and idle, but greatly in debt; and next a morbid consciousness of fraud, and fears of exposure, took possession of his faculties. In this condi- of body and mind he went to bed, where he had remained, without food, for the space of one week, when I first saw him. This was on the 21st of February, at which date I made the following minute of his case:

"'He is very poor in person, and sharp featured; the abdomen is so shrunken that the pulsating aorta can easily be felt; the ribs are drawn down to the crest of the illium; the pulse is very slow, part of the time to forty-six even; the face is turned towards the wall, and the fore-arm, flexed, is laid over the eyes; the respiration is slow and sometimes sighing; the skin is dusky; the tongue clean; the bowels costive; the urine scanty and highly colored. In this state he lies, and it is seldom that his attention can be aroused. When spoken to, he will sometimes draw a long breath and, presently, in a low, half articulate voice, utter a brief reply, but more generally makes no sign of recognition whatever. For the past week he has been under homœopathic treatment in a close chamber. By much persuasion I induced him to take one oyster and two spoonsful of soup, but no more.'

"Viewing the case as one of mental delusion, rather than physical disease, my advice was that the patient should be taken from home, and kept among strangers and strange scenery, till new and rational ideas might take the place of old and delusive ones, after which, I had reason to believe, he would take nourishment and prevent death. I labored a whole week, before I could effect this object.

"At length the reluctance and indecision of friends was overcome, and he was taken on board the cars for New York, on Saturday noon. By my direction, he stopped for the night at Delaware; and there he spent the Sabbath, walking about the streets, talking much of the time. Here he took food also, more than he had done for the week previous. But by his entreaties to return, his son (who unfortunately was his attendant) was prevailed upon to bring him back on Monday. Again I repeated my advice, and urged its adoption, but another physician was called in, who, entertaining different views of the case, thought best that he should remain quietly at home and take medicine. From this time he grew more and more deranged, and took no nourishment, under the obstinate delusion that food, if taken into his stomach, would certainly kill him. During the latter days of his life he moved about more, and was less disposed to maintain silence. Sometimes he would ask for a knife 'to cut open his belly and see what was in it,' 'for a saw to saw his leg off,' also to be 'taken into a field and shot.' These and many other similar expressions, peculiar to the hypochondriac, were made use of by him. He died of starvation, on the 19th of March. I was not present at the post mortem examination, but was informed that no well-marked pathological appearances were discovered, at least none sufficient to account for death.

"Elmira, June 2, 1854."

